# Impact of the COVID-19 pandemic on adults with moderate-to-severe atopic dermatitis in the Dutch general population

**DOI:** 10.1016/j.jdin.2021.12.006

**Published:** 2021-12-28

**Authors:** Junfen Zhang, Laura Loman, Esmé Kamphuis, Marie L.A. Schuttelaar, Lifelines Corona Research Initiative

**Affiliations:** Department of Dermatology, University of Groningen, University Medical Center Groningen, the Netherlands

*To the Editor:* The COVID-19 pandemic might disproportionately impact patients with atopic dermatitis (AD), a chronic inflammatory disorder with immune dysregulation. We conducted a large cross-sectional study to investigate the associations between COVID-19-related impact and AD severity among adults in the Dutch general population.

This study was conducted within the Lifelines Cohort Study,^1^ a multidisciplinary prospective population-based cohort study examining the health and health-related behaviors of 169,729 persons living in the north of the Netherlands in a unique three-generation design. All procedures were approved by the medical ethics committee, and all participants provided written informed consent. AD-related data were collected by sending out a digital questionnaire to all adult participants of the Lifelines Cohort Study (N = 135,950) between February and May 2020 (response rate, 42.4%).^2^ Definitions of AD have been described previously.^2^ COVID-19-related variables were collected by sending out a series of COVID-19 questionnaires (weekly between March and May 2020, biweekly until July 2020, and then at monthly intervals until July 2021) to the adult participant of the Lifelines Cohort Study (N = 139,735),^3^ of those 76,377 (54.7%) responded to at least one questionnaire. The selection of COVID-19 questionnaires varied across outcome measures of COVID-19-related variables. The COVID-19 infection rate, COVID-19 vaccination coverage, and side effects were based on combined answers from all available questionnaires; lung disease, body mass index, smoking habits, and information regarding precautions taken, were collected from the first COVID-19 questionnaire, which was sent out at the same period of AD questionnaire. Quality of health care was collected from the 15th questionnaire, while COVID-19-related psychological impact was collected from the 2nd questionnaire, because only these 2 questionnaires included all the variables related to health care and psychological impact, respectively. Associations between AD severity and COVID-19-related impact were analyzed using binary logistic regression models.

A total of 53,545 participants, who responded to at least 1 COVID-19 questionnaire and responded to the AD questionnaire, were included (Table I). Nonresponders were younger and more often men (data not shown). In the multivariate analysis (Table II), both mild and moderate-to-severe AD showed a positive association with a higher prevalence of lung disease (mild AD: adjusted odds ratio [aOR], 2.50, 95% CI, 1.89-3.30; moderate-to-severe AD: aOR, 3.19, 95% CI, 2.68-3.80). All groups had similar COVID-19 infection rates. Participants with AD, regardless of disease severity, compared with non-AD participants, were more concerned about the COVID-19 crisis (mild AD: aOR, 1.06, 95% CI, 1.00-1.12; moderate-to-severe AD: aOR, 1.08, 95% CI, 1.04-1.12) and more often chose not to contact a doctor when having health problems (mild AD: aOR, 2.52, 95% CI, 1.35-4.67; moderate-to-severe AD: aOR, 2.43, 95% CI, 1.59-3.71). Participants with mild AD, but not moderate-to-severe AD, compared with non-AD participants, had a higher COVID-19 vaccination rate (aOR, 1.44; 95% CI, 1.01-2.05) and more frequently covered their mouth and nose in public (aOR, 1.93; 95% CI, 1.25-3.00). Moreover, only participants with moderate-to-severe AD compared with non-AD estimated a higher chance of becoming infected (aOR, 1.53, 95% CI, 1.00-2.35) and expected a more serious disease course (aOR, 1.51, 95% CI, 1.19-1.91). Those with moderate-to-severe AD compared with non-AD were more worried about getting sick (aOR, 1.41, 95% CI, 1.09-1.83) and a shortage of medications (aOR, 1.34, 95% CI, 1.09-1.65), and they also tended to take other precautions to prevent the spread of the COVID-19 virus (aOR, 1.23, 95% CI, 1.02-1.48). Participants with moderate-to-severe AD compared with non-AD participants, also more often expected side effects (aOR, 1.50, 95% CI, 1.11-2.01) and were more afraid of side effects of COVID-19 vaccines in the short-term (aOR, 1.42, 95% CI, 1.08-1.86) and long-term (aOR, 1.49, 95% CI, 1.19-1.86), and they reported suffering from side effects more frequently (aOR, 1.39, 95% CI, 1.10-1.75).

**Table I:** Characteristics of the participants from the Lifelines cohort, who answered the questions related to AD and COVID-19, stratified for sex∗

	Total, n (%) N = 53,545	Male, n (%) N = 21,021	Female, n (%) N = 32,524	*P* value
Age, y, mean ± SD	55.7 ± 12.5	57.5 ± 12.4	54.6 ± 12.5	**<.001**
Missing, n	0	0	0	
Male	21,021 (39.3)	21,021 (100)	0 (0)	-
Missing, n	0	0	0	
AD prevalence, n (% [95% CI])				
Physician-diagnosed AD in lifetime	4838 (9.1 [8.8-9.3])	1345 (6.4 [6.1-6.7])	3493 (10.9 [10.4-11.1])	**<.001**
Missing, n	489	135	354	
Point prevalence†	1704 (3.2 [3.0-3.3])	534 (2.6 [2.3-2.8])	1170 (3.6 [3.4-3.8])	**<.001**
Missing, n	455	119	336	
Severity prevalence of AD‡, n (% [95% CI])				
Clear or mild	505 (1.0 [0.9-1.0])	191 (0.9 [0.8-1.1])	314 (1.0 [0.9-1.1])	.473
Moderate-to-severe	1188 (2.2 [2.1-2.4])	340 (1.6 [1.5-1.8])	848 (2.6 [2.4-2.8])	**<.001**
Missing, n	458	119	339	
Lung disease (ie, asthma, COPD, chronic bronchitis)	3512 (9.0)	1181 (7.7)	2331 (9.8)	**<.001**
Missing, n	14,438	5730	8708	
BMI, kg/m^2^, mean ± SD	26.1 ± 4.3	26.3 ± 3.6	26.0 ± 4.6	**<.001**
Missing, n	12,398	4992	7406	
Current smoking	3346 (8.1)	1374 (8.5)	1972 (7.8)	**.006**
Missing, n	12,015	4893	7122	
COVID-19 infection and expected disease course				
COVID-19 infection§	2690 (5.1)	948 (4.6)	1742 (5.4)	**<.001**
Missing, n	455	205	250	
Imagine that you get corona, you expect the course of the disease would be (serious complaints/very serious complaints/deadly)	6540 (25.9)	2606 (26.7)	3934 (25.5)	**.030**
Missing, n	28,329	11,256	17,073	
COVID-19 vaccination rate				
At least one vaccine dose against COVID-19‖	27,131 (77.0)	10,516 (77.4)	16,615 (76.7)	.328
Missing, n	18,303	7432	10,871	
Side effects of COVID-19 vaccines				
To what extent the corona vaccine will have serious side effects (often/very often)	2434 (8.5)	795 (7.2)	1639 (9.4)	**<.001**
Missing, n	24,979	9952	15,027	
Afraid of short-term side effects (agree/completely agree)	3163 (11.0)	786 (7.1)	2377 (13.5)	**<.001**
Missing, n	24,918	9937	14,981	
Afraid of long-term side effects (agree/completely agree)	5627 (19.7)	1397 (12.6)	4230 (24.1)	**<.001**
Missing, n	24,918	9937	14,981	
Ever suffered side effects after COVID-19 vaccinations	9845 (40.9)	2707 (29.0)	7138 (48.5)	**<.001**
Missing, n	3078	1172	1906	
Precaution taken				
Frequent hand washing or use of disinfectant	38,866 (95.7)	14,717 (93.2)	24,149 (97.3)	**<.001**
Social distancing	40,115 (98.7)	15,578 (98.6)	24,537 (98.8)	**.048**
Covering mouth and nose in public	1450 (3.6)	543 (3.4)	907 (3.7)	.252
Avoiding the use of public transport	28,223 (69.5)	10,433 (66.0)	17,790 (71.7)	**<.001**
Other precautions	6072 (14.9)	1763 (11.2)	4309 (17.4)	**<.001**
Missing, n	12,920	5223	7697	
Attitudes toward the quality of health care				
It is justified that the capacity for regular health care is reduced in favor of the treatment of corona patients (agree/completely agree)	7525 (26.1)	3406 (30.4)	4119 (23.3)	**<.001**
I am worried that there will be a shortage of medications (agree/completely agree)	7443 (25.8)	2637 (23.5)	4806 (27.2)	**<.001**
The quality of health care is suffering due to the reduced capacity for regular health care (agree/completely agree)	22,022 (76.3)	8678 (77.4)	13,344 (75.5)	**<.001**
More people die as a result of the corona crisis (eg, postponing regular medical treatments, stress, depression) than as a result of the corona itself (agree/completely agree)	15,408 (53.4)	6037 (53.9)	9371 (53.0)	.155
Missing, n	24,668	9815	14,853	
You had health problems that you would normally see the doctor for, but chose not to contact your doctor	753 (2.6)	241 (2.1)	512 (2.9)	**<.001**
Missing, n	24,618	9793	14,825	
Chose not to contact the doctor due to fear of corona	67 (9.1)	24 (10.2)	43 (8.6)	.469
Missing, n	24,634	9799	14,835	
Psychological impact				
Level of concerns about the corona crisis (1-10, mean ± SD)	5.0 ± 2.2	4.6 ± 2.2	5.2 ± 2.1	**<.001**
Missing, n	17,143	6916	10,227	
Quality of life (1-10, mean ± SD)	7.3 ± 1.3	7.4 ± 1.3	7.3 ± 1.3	**<.001**
Missing, n	13,638	5556	8082	
General health (good/very good/excellent)	37,977 (93.7)	14,869 (94.4)	23,108 (93.2)	**<.001**
Missing, n	13,014	5278	7736	
Worry about getting sick (often/always or almost always)	2911 (7.2)	890 (5.7)	2021 (8.2)	**<.001**
Missing, n	13,041	5287	7754	
Estimated chances of becoming infected (high/very high)	865 (3.0)	235 (2.2)	630 (3.6)	**<.001**
Missing, n	25,130	10,133	14,997	

*AD*, Atopic dermatitis; *COPD*, chronic obstructive pulmonary disease; *BMI*, body mass index.

∗ All characteristics are self-reported. Significant *P* values are in bold.

† Determined as the proportion of the participants with self-reported physician-diagnosed AD in a lifetime who had current eczema.

‡ According to the patient-oriented eczema measure, among the participants with self-reported physician-diagnosed AD in lifetime.

§ Defined as receiving either a positive SARS-CoV-2 polymerase chain reaction test or a positive clinician's diagnosis.

‖ The vaccination rate was calculated based on all COVID-19 questionnaires sent out before the end of July 2021. According to the weekly report from the National Institute for Public Health and the Environment, 70% of people of all ages received at least one vaccine dose until July 27, 2021, in the Netherlands.

**Table II:** Impact of the COVID-19 pandemic on adults with AD, stratified for current disease severity∗

	Non-AD in lifetime†, n (%) N = 48,218	Mild AD, n (%) N = 505	Moderate-to-severe AD, n (%) N = 1188	Mild AD vs non-AD		Moderate-to-severe AD vs non-AD	
				Crude OR (95% CI)	Adjusted OR (95% CI)‡	Crude OR (95% CI)	Adjusted OR (95% CI)‡
Age, y, mean ± SD	56.1 ± 12.5	53.4 ± 12.3	50.8 ± 13.0	**0.98 (0.98-0.99)**	**0.98 (0.97-0.99)**	**0.97 (0.96-0.97)**	**0.97 (0.96-0.97)**
Missing, n	0	0	0				
Sex							
Male	19,541 (40.5)	191 (37.8)	340 (28.6)	1	1	1	1
Female	28,677 (59.5)	314 (62.2)	848 (71.4)	1.12 (0.94-1.34)	0.98 (0.79-1.21)	**1.70 (1.50-1.93)**	**1.56 (1.34-1.83)**
Missing, n	0	0	0				
Lung disease (ie, asthma, COPD, chronic bronchitis)							
No	32,470 (92.0)	301 (82.0)	650 (78.3)	1	1	1	1
Yes	2812 (8.0)	66 (18.0)	180 (21.7)	**2.53 (1.93-3.31)**	**2.50 (1.89-3.30)**	**3.20 (2.70-3.79)**	**3.19 (2.68-3.80)**
Missing, n	12,936	138	358				
BMI, kg/m^2^, mean ± SD	26.1 ± 4.2	26.5 ± 4.8	26.5 ± 4.8	**1.02 (1.00-1.05)**	1.02 (0.99-1.04)	**1.02 (1.01-1.04)**	**1.02 (1.01-1.04)**
Missing, n	11,086	128	339				
Current smoking							
No	34,478 (92.0)	350 (91.1)	781 (90.0)	1	1	1	1
Yes	2979 (8.0)	34 (8.9)	87 (10.0)	1.12 (0.79-1.60)	1.03 (0.70-1.50)	**1.29 (1.03-1.61)**	1.13 (0.89-1.44)
Missing, n	10,761	121	320				
COVID-19 infection and expected disease course							
COVID-19 infection§							
No	45,433 (95.0)	473 (94.6)	1103 (94.2)	1	1	1	1
Yes	2390 (5.0)	27 (5.4)	68 (5.8)	1.09 (0.74-1.60)	1.11 (0.71-1.73)	1.17 (0.91-1.50)	1.00 (0.74-1.36)
Missing, n	395	5	17				
Imagine that you get corona, you expect the course of the disease would be							
No or mild complaints	17,046 (74.6)	152 (69.7)	317 (65.0)	1	1	1	1
Serious complaints or very serious complaints or deadly	5795 (25.4)	66 (30.3)	171 (35.0)	1.28 (0.96-1.71)	1.12 (0.79-1.61)	**1.59 (1.31-1.92)**	**1.51 (1.19-1.91)**
Missing, n	25,377	287	700				
COVID-19 vaccination rate							
At least 1 vaccine dose against COVID-19							
No	7156 (22.4)	65 (21.1)	188 (~27.4)	1	1	1	1
Yes	24,645 (77.2)	243 (78.9)	489 (~71.2)	1.09 (0.83-1.43)	**1.44 (1.01-2.05)**	**0.76 (0.64-0.90)**	0.98 (0.79-1.22)
I prefer not to say	111 (0.3)	0 (0)	<10 (~1.5)	-	-	-	-
Missing, n	16,306	197	500				
Side effects of COVID-19 vaccines							
To what extent the corona vaccine will have serious side effects							
Very rarely or rarely or sometimes	23,762 (91.7)	225 (88.2)	468 (86.7)	1	1	1	1
Often or very often	2137 (8.3)	30 (11.8)	72 (13.3)	**1.48 (1.01-2.18)**	1.24 (0.78-1.95)	**1.71 (1.33-2.20)**	**1.50 (1.11-2.01)**
Missing, n	22,319	250	648				
Afraid of short-term side effects							
Completely disagree or disagree or neutral	22,631 (87.2)	211 (~81.2)	438 (~80.5)	1	1	1	1
Agree or completely agree	2768 (10.7)	39 (~15.0)	96 (~17.6)	**1.51 (1.07-2.13)**	1.17 (0.77-1.78)	**1.79 (1.43-2.24)**	**1.42 (1.08-1.86)**
Not applicable	549 (2.1)	<10 (~3.8)	<10 (~1.8)	-	-	**-**	-
Missing, n	22,270	240	640				
Afraid of long-term side effects							
Completely disagree or disagree or neutral	20,471 (78.9)	194 (~74.6)	369 (~67.5)	1	1	1	1
Agree or completely agree	4938 (19.0)	56 (~21.5)	168 (~30.7)	1.20 (0.89-1.61)	1.01 (0.71-1.44)	**1.89 (1.57-2.27)**	**1.49 (1.19-1.86)**
Not applicable	541 (2.1)	<10 (~3.8)	<10 (~1.8)	-	-	**-**	-
Missing, n	22,270	240	640				
Ever suffered side effects after COVID-19 vaccinations							
No	12,794 (58.5)	113 (~51.1)	196 (~44.9)	1	1	1	1
Yes	8713 (39.8)	98 (~44.3)	231 (~52.9)	1.27 (0.97-1.67)	1.17 (0.85-1.61)	**1.73 (1.43-2.10)**	**1.39 (1.10-1.75)**
I don't know or don't remember	360 (1.6)	<10 (~4.5)	<10 (~2.3)	-	-	-	-
Missing, n	2778	20	50				
Precaution taken							
Frequent hand washing or use of disinfectant							
No	1576 (4.3)	21 (5.6)	36 (4.2)	1	1	1	1
Yes	35,057 (95.7)	357 (94.4)	813 (95.8)	0.76 (0.49-1.19)	0.79 (0.50-1.27)	1.02 (0.72-1.42)	0.98 (0.68-1.39)
Social distancing							
No	460 (1.3)	<10 (~2.6)	<10 (~1.2)	1	1	1	1
Yes	36,173 (98.7)	373 (~97.4)	842 (~98.8)	0.95 (0.39-2.30)	4.04 (0.57-28.88)	1.53 (0.72-3.24)	2.29 (0.85-6.16)
Covering mouth and nose in public							
No	35,334 (96.5)	355 (93.9)	822 (96.8)	1	1	1	1
Yes	1299 (3.5)	23 (6.1)	27 (3.2)	**1.76 (1.15-2.70)**	**1.93 (1.25-3.00)**	0.89 (0.61-1.32)	0.92 (0.61-1.40)
Avoiding use of public transport							
No	11,232 (30.7)	113 (29.9)	246 (29.0)	1	1	1	1
Yes	25,401 (69.3)	265 (70.1)	603 (71.0)	1.10 (0.83-1.45)	1.06 (0.84-1.34)	1.08 (0.93-1.26)	1.14 (0.97-1.33)
Other precautions							
No	31,273 (85.4)	318 (84.1)	693 (81.6)	1	1	1	1
Yes	5360 (14.6)	60 (15.9)	156 (18.4)	1.10 (0.83-1.45)	1.10 (0.83-1.47)	**1.31 (1.10-1.57)**	**1.23 (1.02-1.48)**
Missing, n	11,585	120	330				
Attitudes toward quality of health care							
It is justified that the capacity for regular health care is reduced in favor of the treatment of corona patients							
Completely disagree or disagree or neutral	19,338 (73.9)	173 (69.8)	408 (72.7)	1	1	1	1
Agree or completely agree	6826 (26.1)	75 (30.2)	153 (27.3)	1.23 (0.94-1.61)	1.15 (0.84-1.57)	1.06 (0.88-1.28)	0.97 (0.78-1.21)
I am worried that there will be a shortage of medications							
Completely disagree or disagree or neutral	19,513 (74.6)	176 (71.0)	383 (68.4)	1	1	1	1
Agree or completely agree	6650 (25.4)	72 (29.0)	177 (31.6)	1.20 (0.91-1.58)	1.13 (0.83-1.56)	**1.36 (1.13-1.62)**	**1.34 (1.09-1.65)**
The quality of health care is suffering due to the reduced capacity for regular health care							
Completely disagree or disagree or neutral	6287 (24.0)	58 (23.4)	120 (21.4)	1	1	1	1
Agree or completely agree	19,877 (76.0)	190 (76.6)	441 (78.6)	1.04 (0.77-1.39)	0.98 (0.70-1.37)	1.16 (0.95-1.43)	1.13 (0.89-1.44)
More people die as a result of the corona crisis (eg, postponing regular medical treatments, stress, depression) than as a result of corona itself							
Completely disagree or disagree or neutral	12,250 (46.8)	118 (47.8)	265 (47.2)	1	1	1	1
Agree or completely agree	13,910 (53.2)	129 (52.2)	297 (52.8)	0.96 (0.75-1.24)	0.87 (0.65-1.16)	0.99 (0.84-1.17)	0.87 (0.72-1.06)
Missing, n	22,054	257	627				
You had health problems that you would normally see the doctor for, but chose not to contact your doctor							
No	25,581 (97.6)	236 (95.2)	531 (94.5)	1	1	1	1
Yes	631 (2.4)	12 (4.8)	31 (5.5)	**2.06 (1.15-3.70)**	**2.52 (1.35-4.67)**	**2.37 (1.63-3.43)**	**2.43 (1.59-3.71)**
Missing, n	22,006	257	626				
Chose not to contact the doctor due to fear of corona							
No	568 (91.8)	11 (~52.4)	27 (~73.0)	1	1	1	1
Yes	51 (8.2)	<10 (~47.6)	<10 (~27.0)	1.01 (0.13-8.00)	1.16 (0.13-10.69)	1.65 (0.56-4.90)	1.59 (0.49-5.22)
Missing, n	22,018	250	620				
Psychological impact							
Level of concerns about the corona crisis (1-10, mean ± SD)	4.9 ± 2.2	5.1 ± 2.1	5.2 ± 2.2	1.03 (0.98-1.08)	**1.06 (1.00-1.12)**	**1.06 (1.02-1.09)**	**1.08 (1.04-1.12)**
Missing, n	15,482	171	388				
Quality of life (1-10, mean ± SD)	7.3 ± 1.3	7.2 ± 1.3	7.1 ± 1.4	**0.93 (0.86-1.00)**	0.95 (0.87-1.04)	**0.88 (0.84-0.92)**	**0.91 (0.86-0.96)**
Missing, n	12,285	132	332				
General health							
Poor or mediocre	2184 (6.0)	34 (9.1)	112 (13.1)	1	1	1	1
Good or very good or excellent	34,364 (94.0)	339 (90.9)	742 (86.9)	**0.63 (0.44-0.90)**	0.82 (0.53-1.27)	**0.42 (0.34-0.52)**	**0.50 (0.39-0.64)**
Missing, n	11,670	132	334				
Worry about getting sick							
Never or rarely or sometimes	33,963 (93.1)	343 (91.2)	765 (88.3)	1	1	1	1
Often or always or almost always	2534 (6.9)	33 (8.8)	101 (11.7)	1.29 (0.90-1.85)	1.06 (0.68-1.63)	**1.77 (1.43-2.19)**	**1.41 (1.09-1.83)**
Missing, n	11,721	129	322				
Estimated chances of becoming infected							
Very low or low or neutral	24,971 (97.0)	242 (~96.0)	521 (94.0)	1	1	1	1
High or very high	764 (3.0)	<10 (~4.0)	33 (6.0)	0.54 (0.20-1.46)	0.46 (0.15-1.46)	**2.07 (1.45-2.97)**	**1.53 (1.00-2.35)**
Missing, n	22,483	250	634				

*AD*, Atopic dermatitis; *OR*, odds ratio; *COPD*, chronic obstructive pulmonary disease; *BMI*, body mass index.

∗ All characteristics are self-reported. Statistical significance is in bold. If a group size was below 10, we took the following three performances to prevent traceability to particpants: 1) n <10 rather than exact number, was displayed; 2) n <10 was treated as n = 10 when calculating the percentage; and 3) the corresponding number of missing was rounded.

† Based on self-reported physician-diagnosed AD in a lifetime.

‡ Adjusted for age, sex, lung disease, smoking, and BMI.

§ Defined as receiving either a positive SARS-CoV-2 polymerase chain reaction test or a positive clinician's diagnosis.

Our finding of no association between COVID-19 infection rate and the presence of AD in adults is consistent with a recent US study where patients with AD, even those treated with immunomodulatory medications, did not have a significantly elevated risk for COVID-19 infection.^4^ However, COVID-19-related worries were more often seen in patients with moderate-to-severe AD, which might lead patients to practice more precautions in addition to basic rules (eg, hand hygiene, social distance). Furthermore, patients with moderate-to-severe AD tend to encounter dilemmas when comparing the benefit and the potential side effects of COVID-19 vaccines, which may explain why they had comparable vaccination rates to healthy controls. Notably, patients with AD were less likely to search for medical help, reflecting that they did not want to further burden the health care system. Nonetheless, this might also lead to situations where patients miss safety assessments and/or discontinue their treatment, resulting in disease exacerbation, which has been reported in a Danish surveyed-based study.^5^

To summarize, the COVID-19 pandemic has a considerable impact on patients with moderate-to-severe AD, highlighting the need for more attention for their overall wellbeing in daily practice.

## Conflicts of interest

Dr Schuttelaar received consultancy fees from Sanofi Genzyme and Regeneron Pharmaceuticals; and is advisory board member for Sanofi, Regeneron, Pfizer, LEO Pharma, Lilly. Authors Zhang, Loman, and Kamphuis have no conflicts of interest to declare.
